# Early outcomes of MR-guided SBRT for patients with recurrent pancreatic adenocarcinoma

**DOI:** 10.1186/s13014-024-02457-y

**Published:** 2024-05-29

**Authors:** Spencer J. Poiset, Sophia Shah, Louis Cappelli, Pramila Anné, Karen E. Mooney, Maria Werner-Wasik, Talya S. Laufer, James A. Posey, Daniel Lin, Atrayee Basu Mallick, Harish Lavu, Babar Bashir, Charles J. Yeo, Adam C. Mueller

**Affiliations:** 1 Department of Radiation Oncology, Sidney Kimmel Cancer Center of Thomas Jefferson University, 111 S 11th St. Suite G301, Philadelphia, PA 19107 USA; 2 Department of Medical Oncology, Sidney Kimmel Cancer Center of Thomas Jefferson University, Philadelphia, PA USA; 3 Department of Surgery, Sidney Kimmel Cancer Center of Thomas Jefferson University, Philadelphia, PA USA; 4 Department of Radiation Oncology, UPMC Hillman Cancer Center, University of Pittsburgh, Pittsburgh, PA USA

**Keywords:** MR-guided, SBRT, Radiation, Pancreas, Recurrence, Toxicity, Local control

## Abstract

**Background:**

Local treatment options for locally recurrent pancreatic adenocarcinoma (LR-PAC) are limited, with median survival time (MST) of 9–13 months (mos) following recurrence. MRI-guided stereotactic body radiation therapy (MRgSBRT) provides the ability to dose escalate while sparing normal tissue. Here we report on the early outcomes of MRgSBRT for LR-PAC.

**Methods:**

Patients with prior resection of pancreatic adenocarcinoma with local recurrence treated with MRgSBRT at a single tertiary referral center from 5-2021 to 2-2023 were identified from our prospective database. MRgSBRT was delivered to 40–50 Gy in 4–5 fractions with target and OAR delineation per institutional standards. Endpoints included local control per RECIST v1.1, distant failure, overall survival (OS), and acute and chronic toxicities per Common Terminology Criteria for Adverse Events, v5.

**Results:**

Fifteen patients with LR-PAC were identified with median follow-up of 10.6 mos (2.8–26.5 mos) from MRgSBRT. There were 8 females and 7 males, with a median age of 69 years (50–83). One patient underwent neoadjuvant radiation for 50.4 Gy in 28 fractions followed by resection, and one underwent adjuvant radiation for 45 Gy in 25 fractions prior to recurrence. MRgSBRT was delivered a median of 18.8 mos (3.5–52.8 mos) following resection. OS following recurrence at 6 and 12 mos were 87% and 51%, respectively, with a median survival time of 14.1 mos (3.2–27.4 mos). Three patients experienced local failure at 5.9, 7.8, and 16.6 months from MgSBRT with local control of 92.3% and 83.9% at 6 and 12 months. 10 patients experienced distant failure at a median of 2.9 mos (0.3–6.7 mos). Grade 1–2 acute GI toxicity was noted in 47% of patients, and chronic GI toxicity in 31% of patients. No grade > 3 toxicities were noted.

**Conclusions:**

This is the first report on toxicity and outcomes of MRgSBRT for LR-PAC in the literature. MRgSBRT is a safe, feasible treatment modality with the potential for improved local control in this vulnerable population. Future research is necessary to better identify which patients yield the most benefit from MRgSBRT, which should continue to be used with systemic therapy as tolerated.

*Trial Registration*: Jefferson IRB#20976, approved 2/17/21.

## Introduction

Pancreatic cancer is the fourth leading cause of cancer-related deaths in the United States [1]. Its aggressive nature, and late-stage of diagnosis arising from a lack of screening tools pose serious challenges, with resectable pancreatic cancer accounting for about 15–20% of new cases [2]. Currently, surgical resection offers the only potential cure for pancreatic cancer. Unfortunately, despite advancements in surgical techniques and improved adjuvant treatments, the recurrence rate of pancreatic cancer is still remarkably high, approaching 80% following radical resection [3]. For recurrent cases, treatment options typically include re-resection, chemotherapy, radiation therapy, or a combination of both. In patients that do undergo resection and later present with local recurrence of disease, median survival is short, ranging from 9 to 13 months [4–6] on average.

There is a lack of prospective clinical trial data to determine optimal treatment modalities in locally recurrent pancreatic adenocarcinoma (LR-PAC), however re-resection (when feasible) appears to have the best outcomes with a significant survival benefit [3, 7, 8]. Unfortunately, LR-PAC amenable to re-resection is a small subset of the population and requires careful patient selection [7]. A majority of patients are unable to undergo re-resection in LR-PAC, necessitating other local or systemic treatment modalities. Stereotactic body radiotherapy (SBRT) presents an attractive treatment option, achieving a therapeutic benefit with minimal impact on patient quality of life compared to standard fractionated chemoradiation, and is commonly used in the locally advanced, unresectable setting [9, 10]. Meanwhile, reports describing SBRT for LR-PAC are sparse with mixed results on local control with a variety of radiation doses utilized [11–15].

Conventional radiation techniques, including SBRT, struggle to deliver curative doses while sparing nearby radiosensitive abdominal structure due to organ motion, respiratory movement, and limited visibility on CT scans [16]. MRI-guided adaptive stereotactic body radiotherapy (MRgSBRT) holds promise for improving the efficacy and safety of radiation treatment for abdominal malignancies like pancreatic cancer. MRgSBRT offers several advantages over traditional methods. It utilizes pretreatment MRI scans with high soft-tissue resolution, ensuring precise organ and target delineation [17, 18]. Additionally, MRgSBRT enables daily adaptation of treatment plans based on changes in the patient's internal anatomy, with prior retrospective studies demonstrating that adaptation is performed for a majority of treatments and corresponds to increased tumor coverage with down trending dose to organs at risk [17, 19]. Moreover, MRgSBRT uses continuous imaging and target tracking to account for changes in patient's internal anatomy during treatment, allowing for real-time adjustments and treatment pauses if the tumor shifts outside of the planned treatment volume (intrafraction motion) [17, 20]. Breath-hold gating allows for smaller treatment margins without the need for fiducial marker placement. These factors allow for more confidence in treatment delivery and sparing of the normal organs resulting in dose escalation with MRgSBRT to more ablative radiation doses with a higher biologically effective dose (BED_10_) which has been suggested to improve local control and possibly overall survival (OS) in prior studies [21, 22].

The need for improved local therapy in this vulnerable patient population with LR-PAC is evident. MRgSBRT is an ideal treatment modality for a majority of these patients who are unable to undergo re-resection and need effective local therapy that is safe, feasible, and provides durable local control. As pancreatic cancer continues to rise in prominence as a leading cause of cancer deaths in the United States, innovative approaches like MRgSBRT hold the potential to improve these statistics. In this paper, we will review our local control and toxicity results in a small cohort of patients with LR-PAC that underwent MRgSBRT with or without prior radiation.

## Methods

Following institutional review board approval, patients who underwent MRgSBRT for LR-PAC were identified from a prospective database of all patients that have undergone MRgSBRT at the treating facility. Patients were treated with MRgSBRT at a single tertiary referral center from May 2021 to February 2023. LR-PAC was defined as prior resection of pancreatic adenocarcinoma followed by local recurrence of disease at the prior surgical site. Preoperative and post-operative chemotherapy and radiation therapy prior to MRgSBRT were per provider preference. Additional local or systemic therapy following MRgSBRT was per provider preference. Inclusion criteria included: histologic confirmation of pancreatic adenocarcinoma that underwent surgical resection, known extent of resection and pathology results, diagnosis of pancreatic adenocarcinoma ≥ 18 years old, local recurrence of pancreatic cancer treated with MRgSBRT. Exclusion criteria included known metastatic disease prior to or at time of MRgSBRT treatment. One patient with oligometastatic was treated concurrently with MRgSBRT to a single liver lesion in addition to the local pancreatic recurrence. Prior to treatment, all patients were discussed at an intradepartmental MRI LINAC tumor board. A portion of patients were discussed in multidisciplinary tumor board.

MRgSBRT was delivered in 40–50 Gy in 4–5 fractions with target and organ at risk (OAR) delineation per institutional standards. The treatment dose of 50 Gy in 5 fractions and dose constraints in the absence of prior radiation were chosen to match the SMART trial standard [23]. Treatments were normalized up to OAR tolerance to maximize dose but ensure no OAR violated constraints. We aimed for 80% planning target volume (PTV) coverage and 90% gross tumor volume (GTV) coverage to 50 Gy, 95% GTV coverage to 33 Gy, and 95% PTV coverage to 25 Gy. For reirradiation, goals for tumor dosing were reduced to 45 Gy to still achieve a BED_10_ of > 80, while normal tissue constraints were reduced to assume 50% tissue recovery with an α/β of 3 [Table 1].

**Table 1:** Dose constraints for MRgSBRT re-irradiation

Name	Constraint	Target	Priority
Gross tumor volume	V95%[%]	> = 80	2
Optimal planning target volume	Dmin[%]	> = 80	2
Protected planning target volume	V95%[%]	Report	
Planning target volume	V100%[%]	Report	
Body	V65Gy[cc]	< = 0.03	1
Liver	Dmean[Gy]	< = 0	2
Liver	V8Gy[cc]	< = 700	1
Duodenum	V24Gy[cc]	< = 0.5	1
Stomach	V24Gy[cc]	< = 0.5	1
Small bowel	V24Gy[cc]	< = 0.5	1
Large bowel	V24Gy[cc]	< = 0.5	1
Spinal canal	V12.5Gy[cc]	< = 0.5	1
Right kidney	Dmean[Gy]	< = 7	1
Left kidney	Dmean[Gy]	< = 7	1
Right kidney	D66%[Gy]	< = 10	1
Left kidney	D66%[Gy]	< = 10	1

GTV refers to the target tumor volume. PTV is defined as GTV + 3 mm expansion. PTV_Opt is defined as PTV-GI OAR volumes. PTV_High is defined as PTV-PRV 3 mm of GI OAR volumes

Patients were simulated NPO for 3 h and provided a sip of water prior to MRI scan. TRUFI scanning was performed with 17 s breath hold for all patients at comfortable inspiration. CT simulation performed was taken for electron density calculation and isocenter setting. Triple phase diagnostic CT, and when available PET/CT, were fused for target delineation. 3 mm GTV to PTV margins were used, corresponding to a 3 mm gating window, and all treatments were performed with breath hold gating with patient visual feedback. Adaptive replanning was performed for all patients, adaptation was performed for any predicted OAR constraint violation or for 5% or greater reduction in PTV coverage from original plan.

Descriptive analysis of the disease, treatment characteristics, and patients were performed including Karnofsky performance status (KPS) and body mass index (BMI) prior to MRgSBRT. Endpoints included local control and distant failure following MRgSBRT, OS, median survival time (MST) following recurrence, and acute and chronic toxicities per Common Terminology Criteria for Adverse Events (CTCAE), version 5. Local control was defined as absence of tumor progression per RECIST v1.1 criteria. OS was defined as initial histologic diagnosis of pancreatic adenocarcinoma to death. MST following recurrence was defined as time from diagnosis of recurrence to death. Date of recurrence was determined by imaging which had clear evidence of a recurrent mass with MRI abdomen or PET/CT, as re-biopsy was infrequently performed. Descriptive information was gathered from surgical, radiation and medical oncology records. Follow up examinations and imaging were per provider preference.

## Results

Fifteen patients with LR-PAC were identified with a median follow-up of 10.6 months (2.8–26.5 mos) from the time of MRgSBRT. There were 8 females and 7 males, with a median age of 69 years (50–83 years) and a median KPS of 80 (60–100). Fourteen of those underwent surgical resection at the treating radiation facility by two surgeons. Five patients underwent preoperative treatment with four undergoing chemotherapy alone and one patient receiving chemoradiation for 50.4 Gy in 28 fractions followed by surgical resection. Postoperatively, twelve patients underwent chemotherapy, and one underwent chemoradiation for 45 Gy in 25 fractions for positive margins. [Table 2]. MRgSBRT treatment was adapted 94.5% of the time with a median PTV coverage of 86.2% (61.0–99.7) and median GTV coverage of 96.1% (73.5–100) of the prescribed dose. Additional dosimetric information can be found in Table 3.

**Table 2:** Descriptive characteristics and outcomes for all LR-PAC patients that underwent MRgSBRT

	Total (N = 15)
Median age (years)	69.2 (50.4–82.0)
Female	8 (53%)
Histology at initial biopsy	
Ductal adenocarcinoma	15 (100%)
Pathologic staging at initial resection	
IA	1 (6.7%)
IB	5 (33.3%)
IIA	0 (0%)
IIB	3 (20%)
III	6 (40%)
IV	0 (0%)
Median KPS at MRgSBRT	80 (60–100)
Median BMI at MRgSBRT	25.9 (17.8–32.0)
Neoadjuvant chemoRT	1 (6.7%)
Adjuvant chemoRT	1 (6.7%)
Adjuvant chemotherapy without RT	12 (80%)
FOLFIRINOX	5 (33.3%)
Gemcitabine and Capecitabine	3 (20%)
Gemcitabine and Nab-Paclitaxel	1 (6.7%)
Gemcitabine and Cisplatin	1 (6.7%)
Gemcitabine alone	1 (6.7%)
Capecitabine	1 (6.7%)
Median time resection to recurrence (mos)	16.2 (2.6–48.6)
Median time from resection to MRgSBRT (mos)	18.8 (3.5–52.8)
Median follow up from MRgSBRT (mos)	10.6 (2.8–24.8)
Median overall survival (mos)	33.9 (12.4–59.7)
Median survival time from recurrence (mos)	14.1 (3.2–25.6)
Median time to distant failure from MRgSBRT	2.9 (0.3–6.7)
Grade 1–2 GI acute toxicity	7 (46.7%)
Grade 1–2 GI late toxicity	4 (30.8%)

**Table 3:** Dosimetric data of the planning target volume (PTV) and gross tumor volume (GTV) for all MRgSBRT plans

Dosimetric and treatment data	
Percentage of adapted fractions	94.5
Median PTV prescription coverage (%)	86.2 (61.0–99.7)
Median GTV prescription coverage (%)	96.1 (73.5–100)
Median D95% of PTV (Gy)	39.1 (23.5–53.3)
Median D95% of GTV (Gy)	48.2 (29.9–56.2)
Median D99% of GTV (Gy)	39.1 (26.7–55.3)

Recurrence occurred a median of 16.2 months (2.6–48.6 mos) following initial resection, with MRgSBRT being delivered a median of 18.8 months (3.5–52.8 mos) following resection. Dosing and fractionation are described in Table 4.

**Table 4:** Radiation dosing and fractionation for all patients that received MRgSBRT for LR-PAC with biologically effective dose. For reference, conventional radiation for 50.4 Gy in 25 fractions has a BED_10_ of 59.47 Gy

MRgSBRT dosing and fractionation	Biologically effective dose (BED_10_) (Gy)	Total (N = 15)
50 Gy in 5 fractions	100	10 (66.7%)
45 Gy in 5 fractions	85.5	2 (13.3%)
40 Gy in 4 fractions	80	2 (13.3%)
40 Gy in 5 fractions	72	1 (6.7%)

**Fig. 1:**
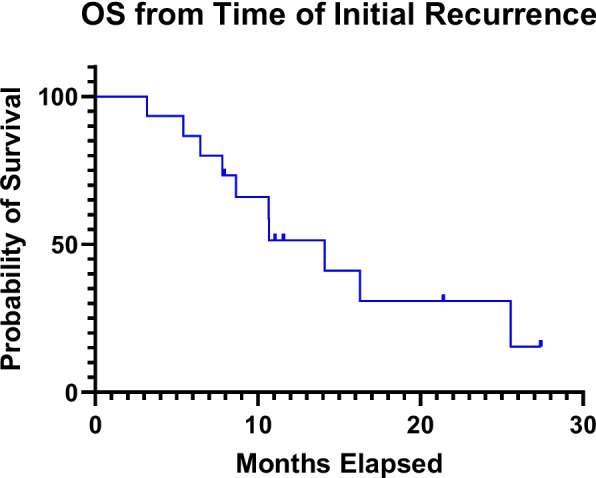
Overall survival of LR-PAC patient from time of initial recurrence. Demonstrates survival curve following diagnosis of initial recurrence for all patients that underwent MRgSBRT for LR-PAC

The OS rates following initial diagnosis at 12, 18 and 24 months were 100%, 87%, and 73%, respectively, with a median OS of 33.9 months (12.4–59.7 mos). OS rates following recurrence at 6 and 12 months were 87% and 51%, respectively, with an MST of 14.1 months (3.2–27.4 months) [Fig. 1]. Three patients experienced local failure first at 5.9, 7.8, and 16.6 months from MgSBRT with a local control of 92.3% and 83.9% at 6 and 12 months, respectively. Meanwhile, 10 patients experienced distant failure at a median of 2.9 months (0.3–6.7 months) following MRgSBRT. Five patients experienced distant failure less than 3 months following radiation. Grade 1 or 2 acute GI toxicity was noted in 47% of patients and chronic GI toxicity, in 31% of patients. No grade ≥ 3 acute or chronic adverse events were noted.

Patients that experienced an earlier recurrence following resection, defined as a recurrence prior to the median recurrence time of 16.2 months, tended to have worse MST following recurrence with 6- and 12-month survival of 71.4% and 28.6%, respectively. While patients with a later recurrence, which occurred after the median recurrence time of 16.2 months, had a survival following recurrence at 6 and 12 months of 100% and 70%, respectively [Table 5].

**Table 5:** Illustrates percentage of the surviving population at 6- and 12-months following recurrence based on tumor and patient specific factors

	6 Month survival (%)	12 Month survival (%)
All patients	87	51
Shorter time to recurrence (< 16.2 mos)	71.4	28.6
Longer time to recurrence (>/= 16.2 mos)	100	72.9
Good Performers at MRgSBRT (KPS >/= 90)	100	80
Poor Performers at MRgSBRT (KPS </= 80)	80	35
Obese (BMI > 24.9)	100	75
Normal or underweight (BMI < 25)	71.4	21.4
Chemotherapy at time of recurrence	100	100
No chemotherapy at time of recurrence	83.3	38.1

Other factors that seemed to be associated with improved survival following recurrence were performance status, BMI, and treatment with chemotherapy at time of recurrence [Table 4]. Patients with KPS of 90–100 at time of recurrence had 12-month survival following recurrence of 80% compared to 31.1% in poorer performers with KPS of 60–80. Obese patients at the time of recurrence had a 6- and 12-month survival of 100% and 71.4% as compared to normal or underweight patients with 6- and 12-month survival of 66.7% and 25%, respectively. All 3 patients that underwent additional chemotherapy at time of recurrence were alive at 12 months, compared to 33.3% 12-month survival in the 12 patients that did not.

## Discussion

Even with optimal patient selection, tumor characteristics, and treatment considerations, a majority of patients with initially resected pancreatic cancer have disease progression with either locoregional and/or distant failure. A retrospective review from the Johns Hopkins Hospital demonstrates 76.7% of patients had recurrence, with 23.7% developing local recurrence only [24]. Examination of patterns of local recurrence from ESPAC-4, a large multicenter clinical trial, demonstrated 65.6% disease recurrence of the 730 patients that underwent initial resection, with only local recurrence in approximately half of patients with recurrence [5]. These results emphasize both the need for improved up-front treatment paradigms, as well as effective tools for the treatment of recurrences when they occur.

Local control is crucial in pancreatic cancer, as evidenced by published data indicating that 30% of patient deaths result from local disease progression [25]. There are no clear guidelines for the treatment of LR-PAC, with re-resection, chemotherapy alone, chemoradiation, and SBRT all being potential therapies. There is a lack of prospective clinical trial data to determine optimal treatment modalities, however re-resection appears to have the best outcomes with a significant survival benefit [3, 4, 7, 8, 26]. A systematic review by Groot et al. identified the best survival outcomes with re-resection at 32 months, while chemoradiotherapy and SBRT are similar at 19 and 16 months, respectively [4]. Reoperation is a safe an effective option in a carefully selected group of patients [26]. That being said, patients with LR-PAC that are amenable to re-resection make up a small subset of the population. Chemoradiation for LR-PAC as a means to re-resection has also been explored previously with Habermehl et al. reporting on retrospective outcomes in 41 patients undergoing fractionated chemoradiotherapy. Treatment was well tolerated, however only 12% were able to undergo re-resection and only 15% had complete response with chemoradiation further highlighting the need for improved local therapy in LR-PAC [7].

SBRT is an attractive treatment option given that it can be completed quickly while achieving biologic equivalent doses similar to long course chemoradiation [9]. The majority of published data regarding SBRT for pancreatic cancer pertains to locally advanced or unresectable disease, with SBRT for LR-PAC being sparse. A small study by Reddy et al. demonstrated in a cohort of 19 radiation naïve patients with LR-PAC, that SBRT is safe and feasible with a mean BED_10_ of 54.8 Gy. Despite relative safety in this challenging patient population, 1 patient experienced gastric perforation and almost 50% experienced local failure, with BED < 54.8 Gy being associated with inferior local control [13]. Several other small cohorts of patients treated with SBRT for LR-PAC have been reported on with variable SBRT dosing and local control outcomes. A few studies reporting on treatment with conservative SBRT doses to a median 24 or 25 Gy had good toxicity outcomes, however they demonstrated relatively poor local control of 56–78% at 6 months and 19–72% at 12 months [11, 14, 15]. Meng et al. had the highest treatment dose for SBRT to a median of 45 Gy (42–50 Gy) for 19 patients, with the best reported local control at 6 months of 95%, although control declined significantly to 45% at 12 months [12]. Despite variation among the trials in patient population, prior therapies, and SBRT dosing for LR-PAC, overall survival did not vary significantly among these trials ranging from 9 to 13 months [11, 12, 14, 15]. Although the local control of 83.9% at 1 year for LR-PAC treated with MRgSBRT is an improvement from prior studies of SBRT for LR-PAC, it similar to published data for borderline and unresectable primary disease with studies demonstrating a local control of 82.4–82.9% [22, 23].

The results from our small cohort of LR-PAC that underwent MRgSBRT demonstrate excellent safety and toxicity, with no grade 3 toxicities even among patients with prior radiation. This is comparable to prior published data on SBRT in LR-PAC, even when compared to much more conservative doses in non-MRgSBRT [11, 13–15]. Although overall survival with our patient cohort is similar to prior literature, this is unsurprising. Even among prior SBRT data for LR-PAC, the survival following initiation of SBRT varies significantly. Interestingly, in examination of recurrence pattern in ESPAC-4, there was no significant difference in overall-survival between local and distant recurrence following disease recurrence, with median survival of 9.5 and 9.4 months, respectively. There was also no significant difference in overall survival between local and distant recurrence from initial resection, despite distant recurrence occurring earlier than local recurrence [5]. This highlights the fact that in most patients pancreatic cancer is a systemic disease and adequate multimodality therapy with local and systemic therapy is needed to appropriately treat patients.

Although local control is important, there are several factors that influence survival following recurrence including tumor and patient specific factors. This is supported by our data which shows improved survival from recurrence in good performers (KPS > 90), overweight (BMI > 24.9) compared to normal and underweight patients, patients able to receive additional chemotherapy at the time of recurrence, and longer time to recurrence from initial resection. The observation that overweight patients have better survival is likely due to the fact that many of the normal and underweight weight patients had significant weight loss prior to presentation as compared to people that maintained weight with BMI > 24.9. Both BMI and ability to receive chemotherapy are similar markers to performance status.

Importantly, with the ability to dose escalate and adequately cover the target volume with MRgSBRT it seems to confer a local control benefit as compared to non-MRgSBRT, with a 6- and 12-month local control of 92.3% and 83.9%, respectively. The 3 cases of local failure in our population could be explained by pancreatic adenocarcinoma’s inherent radioresistance, but it is also likely that portions of tumor were under dosed in order to protect normal organs.

A strength of the study includes its reporting on novel treatment modalities with MRgSBRT, as well as capturing data in a prospective manner. Limitations of the study include its small population size as well as reporting on patients treated at a single academic center. Additionally, as it captures a wide variety of patients with a range of prior therapies, disease stage, treatment sites and radiation dose, precise interpretation of the results remains difficult.

## Conclusions

This is the first report on toxicity and outcomes of MRgSBRT for LR-PAC in the literature. Despite surgery being the only potentially curative therapy for pancreatic cancer, a majority of patients that undergo resection experience local and/or distant recurrence. In the setting of LR-PAC, treatment guidelines remain obscure with only a small portion able to undergo re-resection. Despite the small sample size, our results suggests MRgSBRT is a safe and feasible treatment modality with a potential for improved local control in this vulnerable population. Future research will be necessary to better identify which patients yield the most benefit from MRgSBRT, which should be continued to be used with systemic therapy as tolerated.

## Data Availability

The datasets used and/or analyzed during the current study are available from the corresponding author on reasonable request.

## Abbreviations

LR-PAC: Locally recurrent pancreatic adenocarcinoma
SBRT: Stereotactic body radiotherapy
MRgSBRT: MRI-guided adaptive stereotactic body radiotherapy
BED_10_: Biologically effective dose
OS: Overall survival
MST: Median survival time
CTCAE: Common Terminology Criteria for Adverse Events
BMI: Body mass index
KPS: Karnofsky performance status
